# Chemo-Radio-Immunotherapy Strategies to Prevent Immune Resistance in Non-Small Cell Lung Cancer

**DOI:** 10.3390/cancers18010137

**Published:** 2025-12-31

**Authors:** Renata-Andrea Rusu-Patraulea, Petronela Rusu, Tudor-Eliade Ciuleanu

**Affiliations:** 1 Faculty of Medicine, University of Medicine and Pharmacy “Iuliu Hatieganu”, 8 Victor Babes Street, 400012 Cluj-Napoca, Romania; andreapatraulea@gmail.com (R.-A.R.-P.);; 2 Radiotherapy Laboratory, Oncology Institute “Prof. Dr. Ion Chiricuta”, 34-36 Republicii Street, 400015 Cluj-Napoca, Romania

**Keywords:** chemo-radio-immunotherapy, immune resistance, NSCLC

## Abstract

Immunotherapy (IT) and especially immune checkpoint blockade (ICB) changed the therapeutic approach in non-small cell lung cancer (NSCLC). Nevertheless, primary or secondary resistance and a percentage of long responders and survivors have been observed. The aim of this study is to gain a deeper understanding of the complex mechanisms of primary and secondary resistance to IT, involving tumor cells, the tumor microenvironment (TME), and the host, in order to find strategies to overcome it. With this aim in mind, a search for key words has been performed to identify relevant evidence in the literature. The most widely used approach is the combination of IT with chemotherapy (CT) and/or radiotherapy (RT), relying on the synergistic effect on the enhancement of immunogenic cell death. Since a dual role has been observed, a lot of questions are yet to be answered regarding the complex effect of these therapies, especially on the TME. Preclinical and clinical studies investigate the best sequencing and timing of chemoradiation with IT, and the optimal RT volumes, sites, and dose/fractionation regimens to favor immune stimulation over suppression on the TME. Moving forward, multiple agents addressing coinhibitory or costimulatory receptors on immune or tumor cells are under evaluation. The huge potential of combination therapies becoming apparent. Questions regarding targets, selection of patients, and time and sequence of administration are yet to be answered, considering the complex mechanisms of resistance. Dynamic biomarkers to guide personalized treatment decisions are needed.

## Simple Summary

Immunotherapy and especially immune checkpoint blockade changed therapeutic approaches in non-small cell lung cancer. Nevertheless, primary or secondary resistance and a percentage of long responders and survivors have been observed. The aim of this study is to gain a deeper understanding of the complex mechanisms of primary and secondary resistance to immunotherapy in order to find strategies to overcome it. The most widely used approach is the combination of immunotherapy with chemotherapy and/or radiotherapy, relying on the synergistic effect on the enhancement of immunogenic cell death. However, a dual role has been observed, considering in particular the complex effect on the tumor microenvironment. Preclinical and clinical studies investigate the best sequencing of chemoradiation with immunotherapy and the optimal radiotherapy volumes, sites, and dose/fractionation regimens; dynamic biomarkers are necessary in order to guide decisions. Moving forward, multiple agents addressing coinhibitory or costimulatory receptors on immune or tumor cells are under evaluation.

## 1. Introduction

In the last decade, IT and especially immune checkpoint inhibitors (ICIs) have revolutionized cancer treatment and changed the therapeutic strategies in lung cancer, the most frequently diagnosed cancer, with 2.5 million new cases per year and the leading cause of death, with an estimated 1.8 million deaths per year [1].

Cancer IT has a history of more than 130 years, going back to William B. Coley, who is considered its promoter, as he observed in 1891 that a patient with sarcoma presenting post-surgery erysipelas infection with streptococcus pyogenes experienced a complete regression of his cancer. He explained this remission as immune reaction and started to treat cancer patients with bacterial products [2]. A century of controversies followed, until the interest in the role of the immune system in cancer patients reemerged and led to the birth of immuno-oncology [3].

### 1.1. Cancer Immune Surveillance and Immunoediting

In the 1950s, a hypothesis of “cancer immune surveillance” was elaborated independently by M.F. Burnet and L. Thomas, claiming that the immune system can recognize tumor antigens and can eliminate tumor cells. This theory raised a lot of debate, but it was only in 2001 that R.D. Schreiber and colleagues provided evidence that chemically induced sarcoma grew better in immune-incompetent mice than in wild animals. Furthermore, they showed that 40% of chemically induced tumors from immune-incompetent mice were rejected when transplanted to immune-competent hosts, due to T cell-mediated immune responses followed by immunologic memory [3].

Finally, it was recognized that immune surveillance represents only one dimension of the complex interaction between the immune system and cancer, inducing a new concept of cancer immune editing and “the three Es” theory, which assumes three phases: elimination, equilibrium, and escape [3,4]. In the first phase of elimination or immune surveillance, the immune system is capable of eradicating the developing tumor cells. A cancer immune cycle was described in seven steps by Daniel S. Chen and Ira Mellman in 2013 [5]. Antigens released by tumors are taken over by dendritic cells. In the process of priming, which takes place in the lymph node, these antigens are presented to the T lymphocytes, which undergo activation and clonal expansion. Traveling through blood vessels, and passing between endothelial cells, drives the infiltration of T cells into the tumor, where they develop the ability to recognize and to destroy tumor cells and produce more antigens to augment the process. This elimination phase is considered immunogenic cell death.

The process is more complex, with several interactions between the tumor cells, their immunogenic capacity, mutational load, sensitivity to the immune effector, and the general immune status of the host, characterized, for example, by lymphocyte count, infiltration with T cells, and the presence or absence of immune checkpoints [6].

Despite these interactions, spontaneous tumor regressions are rare because of progressive developments of inhibition mechanisms of the immune system. For a long period of time, which can be several years, an equilibrium state is developing between immune surveillance and the inhibition mechanisms of the immune system, but still with the capability of controlling the disease. Following genetic instability and immune selection, new variants of tumor cells are immunologically “sculpted by immune editors”. Finally, these variants of tumor cells manage to escape from immune surveillance and proliferate by becoming clinically detectable.

### 1.2. Immune Checkpoint Blockade (ICB)

With the aim of better understanding the process of immune evasion, one of the first mechanisms proved was the direct inhibition of T cells. James P Allison and Tasuku Honjo discovered the immune checkpoint function of receptors like Cytotoxic T Lymphocyte Associated Antigen 4 (CTLA-4) and Programmed Death 1 (PD-1) on the surface of T cells, acting like “brakes” of the immune system, preventing autoimmune reactions. For their insights regarding cancer therapy by inhibiting negative immune regulation via the antibody blockade of these receptors, they received the Nobel Prize in Physiology and Medicine in 2018. Based on clinical benefits, the US Food and Drug Administration (FDA) approved several monoclonal antibodies (mAbs) like ipilimumab-targeting CTLA-4, first in melanoma, then in lung cancer; nivolumab and pembrolizumab targeting the receptor PD-1 on T cells; and atezolizumab and durvalumab targeting the ligand PDL-1 on lung tumor cells, thus preventing binding to the PD-1 receptor on the T cell. The first PD-1 inhibitor, nivolumab, was approved in 2014 for previously treated advanced squamous and non-squamous NSCLC, following the results of CheckMate 017 and CheckMate 057 trials [7,8,9].

In the meantime, the combined long-term results of the two trials showed significant benefit for nivolumab, with a 5-year overall survival (OS) of 13.4% compared to 2.6% for the standard second-line treatment with docetaxel [10]. The results proved to be even better for patients with PD-L1 expression ≥ 1%, obtaining 18.3% vs. 3.4%.

## 2. Primary and Secondary Resistance

Despite these unprecedented results, the survival curves reveal in the first 6 months an overlapping steep part followed by a progressive separation of the curves, with leveling after 3 years for the nivolumab arm, obtaining a certain percentage of long-term survivors. Progression-Free Survival (PFS) curves also overlap in the first 6 months, reaching about 40% of patients without disease progression. The initial steep part of the nivolumab curve can be interpreted as an intrinsic, primary resistance, meaning a lack of initial response, whereas the second part represents an acquired, secondary resistance, translated into disease progression after an initial response to IT. Of the patients, 60% responded at 1 year and about 40% had progressive disease [10]. The main question is how to identify these categories of patients, including the long-term survivors.

In the OAK trial, amongst patients who received one or two previous platinum-based CT regimens for stage IIIB or IV NSCLC with atezolizumab in second- or third-line treatment versus docetaxel, progressive disease (PD) was reported in about 44% [11].

In the Keynote 024 trial, in first-line treatment with pembrolizumab versus platinum doublet in advanced NSCLC, PDL1 ≥ 50%, and no sensitizing mutation of the epidermal growth factor receptor gene (EGFR) or translocation of the anaplastic lymphoma kinase gene (ALK), after overlapping curves in the first 3 months, PFS at 6 months was 62% vs. 50.3%, meaning that 38% of patients were in progression or deceased [12].

In the Keynote 042 trial, patients who had previously untreated advanced NSCLC with PD-L1 expression in at least 50% of tumor cells and EGFR, ALK negative, were randomized to receive in first-line treatment either pembrolizumab or the investigator’s choice of platinum-based CT. The objective response (OR) was higher in the pembrolizumab group than in the CT group, 44.8% vs. 27.8%, and 21% of patients progressed [13].

Zhou S. et al. [14] emphasized that primary resistance to IT in NSCLC patients is around 21–27% for nivolumab alone or combined with another ICI like ipilimumab as first-line treatment [15,16], and 40–44% in second-line treatment. When combining IT with CT as a first-line treatment, the incidence of primary resistance dropped to 9–11% [17,18,19]. Secondary resistance to IT in first-line treatment was 52% in the Keynote 042 study, and in second-line treatment was 55% in the OAK study and 64% in the pooled analysis of the CheckMate 017 and CheckMate 057 trials, at four years of follow-up.

In April 2019, the Society for Immunotherapy of Cancer (SITC) developed expert consensus definitions for the PD-L1 inhibitor resistance, including clinical definitions of primary resistance, secondary resistance, and resistance that develops after discontinuation of therapy [20].

Despite no uniform agreement on every issue, primary resistant disease was considered in a patient who has PD, or stable disease (SD) less than 6 months, after receiving at least 6 weeks of exposure to PD-L1 checkpoint inhibitors, but no more than 6 months. RECIST1.1 was used as response criteria, in addition to immune RECIST (iRECIST) for mixed responses and pseudo progression. However, evaluating diameters of tumors might not be accurate in many circumstances, as an excavating tumor is witnessed often after IT. On the other hand, caveats to primary resistance are late responders after initial progression, with frequency varying between drugs and tumor types. Also, treatments beyond progression are reasonable, especially in oligo-progression, where local therapy to the progressing lesion can be administered. Patients who experience early toxicities, needing steroids and cessation of PD-L1 inhibitors, are difficult to assess for primary resistance.

Secondary resistance can be considered when a patient treated with PD-L1 checkpoint inhibitors has a documented, confirmed OR, or prolonged SD of more than 6 months, and then has PD. Reconfirmation by imaging procedures is needed within 4–12 weeks after the first evidence of PD, in the setting of ongoing treatment, although the timeframe may need to be rethought in indolent tumor types. All definitions are to be based on patients being treated with systemic anti- PD-L1 monotherapy, and combinations with other ICIs and other types of systemic or local therapies were not addressed [20].

## 3. Mechanisms of Resistance

Resistance mechanisms can be attributed to tumor cells, immune cells, and the host [21]. Primary resistance may result mainly from low tumor immunogenicity, impaired antigen presentation, and an immunosuppressive TME, whereas the complex mechanisms of secondary resistance involve tumor cells and the TME. Tumor cells are characterized by heterogeneity and may gain clonal neoantigen depletion. The impact of ICB might be a sub-clonal selection of tumor cells with genomic and epigenetic transformation, responsible for immune inhibitory signals. Immune cells can undergo exhaustion or dysfunctions, responsible for defective priming and immune-suppressive signals, with the polarization of the TME cells towards immune-suppressive cells like myeloid-derived suppressive cells (MDSCs), regulatory T cells, (T regs), type 2 macrophages (TAM2) converted from type 1 macrophages (TAM1), or type 2 neutrophils (TAN2) from type1 neutrophils (TAN1), and cancer-associated fibroblasts (CAFs). Host factors can consist of altered antigen recognition due to HLA loss and defective metabolic adaptation or cytokine production. Microbiomes, diet, and medication like antibiotics and steroids can also be encountered as factors.

Mechanisms of resistance can also be classified according to each phase of the cancer immunity cycle. Firstly, a low tumor mutation burden (TMB) and a reduction in neoantigens can take place, affecting tumor immunogenicity. Next-generation sequencing applications have found that gene mutations affecting TMB and PD-L1 expression may be involved in primary and secondary acquired resistance to IT. For example, in oncogene driver mutated subsets of NSCLC cells like EGFR, HER2 or ALK, ROS, RET and MET fusions, limited benefit was obtained from ICB, despite enhanced PD-L1 expression, possibly because of downregulation of the TMB and tumor-infiltrated lymphocytes (TILs) [14,22]. Thus, the most applied biomarkers, TMB and PDL1 expression, seem to be independent factors influenced by the complex mechanisms of resistance involved.

Secondarily, impaired recruitment and dysfunction of DCs with antigen processing and presentation have been encountered. Human Leukocyte Antigen (HLA) gene loss affects neoantigen presentation [14,23], leading to an impaired priming or activation of the T cells. In 40% of cases, NSCLC cells are characterized by allele-specific HLA loss, related to high TMB and positive PD-L1, but poor response to ICIs [24,25].

Recent studies have shown that mutations in KRAS, SKT11, KEAP1, JAK1/2, B2M, APC, MTOR, and TP53 and co-mutations of these genes are the main determinants of mechanisms of resistance to ICIs in NSCLC patients. These mutations produce epigenetic changes responsible for metabolic changes in cancer cells and cytotoxic T cells [26,27].

Downregulation of chemokines such as CXCL9 and CXCL10 inhibits T cell trafficking, while upregulation of VEGF and TGF-β prevents T cell infiltration into the tumor. These processes promote an immune-desert or immune-excluded tumor microenvironment, ultimately impairing tumor cell elimination. This is further reinforced by the upregulation of immunosuppressive cells and cytokines, along with the co-expression of multiple immune checkpoint molecules [21].

The main objective consists of overcoming resistance mechanisms to IT. In this regard, combination treatments might be the reasonable answer. However, it is unlikely to be able to target all of these mechanisms simultaneously. The most accessible approach is the combination of CT and RT with IT.

## 4. Preclinical Studies Combining Immunotherapy with Chemotherapy and Radiotherapy

Evidence from preclinical studies underlined a synergistic anti-tumor effect, by combining IT with CT and RT, ensuring increased cytotoxicity, enhancement of immunogenic cell death, tumor necrosis, and neoantigen creation, thus providing the multi-modality treatment strategy. However, a double-edged effect has been described [2,6,7].

### 4.1. Chemotherapy

CT demonstrates great value in combination with IT. Despite its immunosuppressive effect on the bone marrow, it is responsible for the induction of immunogenic cell death by direct cytotoxicity, producing lethal and sub-lethal DNA damage, ensuring neoantigen production, and also providing immunoregulatory functions by enhancing APC and effector T cell response and disrupting immune-suppressive pathways. The most frequently used drugs in NSCLC are platinum compounds, cisplatin or carboplatin, etoposide, and third-generation drugs like vinorelbine, paclitaxel, and pemetrexed, the latter used in non-squamous NSCLC [7,28,29,30,31].

Platinum compounds promote upregulation of major histocompatibility complex (MHC) class I expression, release of tumor antigens, emission of danger-associated molecular patterns (DAMPs), recruitment and proliferation of effector cells, upregulation of cytotoxic effectors, and downregulation of the immune-suppressive microenvironment.

Whereas the immunomodulatory role of etoposide is not fully understood, with suppression of inflammatory cytokine levels and apoptosis of peripheral activated lymphocytes [17], Gameiro et al. showed that vinorelbine, a semi-synthetic vinca alkaloid which interferes with the polymerization of tubulin, contained in microtubules, thus blocking cell division, in combination with cisplatin, enhances MHC class I expression, increases sensitivity to perforin/granzyme-mediated cytotoxic T Lymphocyte killing by modulation of tumor phenotype, and affects cytokine/chemokine expression and the proapoptoic/antiapoptoic gene ratio, such as tumor necrosis factor-α (TNFα), IL8, CXCL5, and B cell lymphoma-2 gene (BCL-2). The data are complementary to that of immunogenic cell death, suggesting the benefit of combining CT with IT [32].

Paclitaxel, naturally produced in the bark and needles of Taxus brevifolia, increases the rate of apoptosis in tumor cells, releasing tumor antigens, thus enhancing the antigen presentation and phagocytosis of the APCs. Furthermore, paclitaxel increases the proinflammatory cytokines such as IL-10 and decreases the number and activity of T regs [33].

Pemetrexed, used in non-squamous NSCLC, inhibits folate metabolism and stimulates the activity and infiltration of T cells, making it a candidate for combination with radiotherapy and IT [34].

### 4.2. Radiotherapy

Preclinical studies have proven an immune activation by RT, through direct DNA damage or by generation of free radicals. Also, it induces the release of DAMPs, producing a series of biological events like neoantigen generation, APC recruitment and antigen presentation, priming and T cell proliferation, and trafficking and infiltration of the tumor. Finally, it enables T cells to recognize and destroy tumor cells in the irradiated volume or even at distant non-irradiated sites, triggering systemic antitumor response, through infiltrating immune cells and reprogrammed TME and alteration of cytokines and chemokines, inducing the abscopal effect. This is more frequently observed when irradiation is combined with IT [2].

Frey et al. [35] demonstrated that hypo-fractionated RT of two fractions of 5 Gy induced a significant infiltration of APCs on days 5–8 in colorectal cancer. CD8+ T cells recruited by activated APCs were enhanced on day 8. As the immune cell infiltration takes place in a narrow window of time, it can be hypothesized that the effect of RT can be boosted by IT. Furthermore, the infiltration of immune-suppressive cells such as T regs and MDSC is less influenced by hypo-fractionated RT. There is a slight increase in T regs on day 8–10, following the infiltration of cytotoxic T cells, suggesting that reirradiation of the tumor on day 9–10 would be optimal. RT also impacts the recruitment and activation of APCs and T cells according to dose, at an administration of 2 Gy/fraction but even more at 5–8 Gy/fraction. Vanpouille et al. showed that three doses of 8 Gy produced accumulation of double-strand DNA fragments in the cytosol and activation of INF-I pathway via cyclic GMP-AMP (cGAMP) synthase (cGAS). The downstream stimulation of interferon genes (STING) produces INFβ, and finally activates DCs and priming of tumor-specific CD8+ T cells. Meanwhile, single-fraction, high-dose treatment with a threshold between 12 and 18 Gy, induces activation of exonuclease Trex1 at levels that degrade the accumulated DNA fragments in the cytosol of irradiated tumor cells, preventing the INF-I pathway. These data have an important implications for the choice of radiation dose and fractionation in the clinic, to convert unresponsive patients into responders to IT [36].

Dovedi et al. [37,38] obtained better survival curves when RT 5 × 2 Gy was administered concurrently with an PD-L1 antibody on day 1 or 5, than sequentially on day 7. Finally, Demaria et al. [39] demonstrated the abscopal effect, the reduction in tumor growth outside the field of radiation, in mice who received RT and growth factor Flt3-Ligand (Flt3-L), but not Flt3-L or RT alone, or in mice with T cell deficiency, emphasizing their role in this process. Moreover, her group demonstrated that fractionated, but not single-dose radiotherapy induces an immune-mediated abscopal effect when combined with anti-CTLA-4 antibodies [40].

Also, studies in murine lymphoma models demonstrated that the combination of RT with stimulatory CD40 mAbs produced an enhancement of DC functions, through increased expression of MHC molecules and proinflammatory cytokines finally stimulating T cell trafficking and M1 polarized macrophages [41,42].

RT also produces an activation of mTOR signaling, as part of the DNA damage response, leading to an increase in peptide presentation by tumors and T cell activation [43,44].

On the other hand, preclinical studies have also demonstrated immune suppression by RT on circulating lymphocytes B or T and lymphoid tissue, which are highly sensitive to irradiation, whereas CD4+ T cells or T-regs are more resistant, and a slight increase was obtained on days 8 to 10 of the latter [35]. Deng et al. [45] showed growth of PD-L1 expression after RT. Derer et al. [46] suggested a tumor cell-mediated upregulation of PD-L1 expression following chemoradiation, dependent on fractionation of RT, offering a good reason for combination with ICIs. Furthermore, the dual role of RT on the TME has been emphasized. Zang et al. described in detail the immunostimulatory effect, produced by damaged DNA on the cGAS-STING and Type I interferon signaling, activating the DCs and priming the T cells. On the contrary, immunoinhibitory effects on tumor cells and stromal and immune cells are reprogramming the TME. To influence this balance, various complex interactions and modulations of the secretion of chemokines and cytokines or growth factors take place [2]. For example, DCs migration is favored by chemokines CCL7 and CCL21. T cell priming, CD8+Tcell cytotoxicity is mediated by interleukin IL-12, NK enhancement by IL-18, TAM1 polarization by TNFα, and TAN1 polarization by INFβ. On the other hand, immunoinhibitory effects like TAM2 infiltration and differentiation are favored by CXCL2, CXCL12, CCL2,3,5 and MDSC and Tregs recruitment by CCL2 and CCL22, 28, respectively. IL-10 promotes APC inhibition, Treg action, and IL-1 induction of MDCs. Growth factors like colony stimulating factor 1 (CSF1) favor TAM mobilization and proliferation and MDCs recruitment. G-CSF induces neutrophiles mobilization, whereas VEGF produces Treg proliferation. TGF-β favors TAM2 and TAN2 polarization, CD4+ T cell transformation in Tregs, and NK suppression.

### 4.3. Ongoing Questions for Clinical Studies

A series of ongoing questions arose from preclinical studies and ought to be verified in clinical trials. The main question is regarding the optimal RT schedule to elicit an immune response, to transform a tumor from “cold to hot” and stimulate the TME, or to prevent a polarization of TME cells into immunosuppressive cells. Regarding the best dose per fraction, preclinical studies suggested the classical 2 Gy/fraction. Higher doses like 5–8 Gy/fraction have proved to be even better, but not a single-fraction high dose of 12–18 Gy. This could have critical implications for clinical translation, especially in SBRT doses per fraction higher than 12 Gy.

Concerning the number of fractions, the clinical abscopal effect was observed following 3–5 fractions, whereas protracted RT may induce more lymphopenia. Also, questions arose about the total duration of RT and the timing with the IT, and whether it should be concurrently or sequentially.

Other questions are linked to the optimal RT target as to whether there should be single or multiple targets, the primary tumor or metastases; whether one or all metastases should be irradiated, taking into account the sub-clonal neoantigens; and regarding the abscopal effect [47]. We reported an abscopal effect after hypo-fractionated RT, 20 Gy/5 fractions of the painful metastases on the 7th left rib and concomitant nivolumab, in a patient with stage IV adenocarcinoma of the lung who progressed after first-line CT, with two paraaortic lymph nodes and metastases on the 10th and 7th left ribs. After 26 cycles, a complete response was revealed on PETCT and maintained for 77 cycles and beyond, observed via clinical and imagistic follow-up for five years [48].

There are also questions about how many lymph nodes should be irradiated, considering the sensitivity of lymph nodes and circulating lymphocytes.

With the advent of the association with radio-immunotherapy, the philosophy of irradiation towards deescalation of the doses and definition of target volumes would be changed. Most likely, the clinical target volume would be reduced as much as possible, as large volumes would cover more lymphoid tissue. Besides these questions of modulation of dose, time, and volume, the sequencing of therapies and type of CT and IT are parameters that require further investigation in clinical studies [47].

## 5. Clinical Studies

After the good results of Pembrolizumab versus CT in the Keynote 024 and 042 studies for patients with PD-L1 expression ≥ 50% [12,13], the combination of pembrolizumab with different CT regimens in the Keynote 189 and 407 trials demonstrated a further significant improvement of patients’ survival compared to CT alone, regardless of PD-L1 status. These combinations became the standard of care in the guidelines for advanced non-squamous and squamous lung carcinoma, with good performance status and without oncogenic driver mutations. The Keynote 189 trial for non-squamous NSCLC [49] compared pembrolizumab versus placebo for up to 35 cycles, associated with pemetrexed and a platinum compound for four cycles, followed by maintenance pemetrexed, obtaining a 5y-OS of 19.4% versus 11.3%. The Keynote 407 trial for squamous NSCLC [19] compared pembrolizumab versus placebo for up to 35 cycles, with carboplatin and paclitaxel, or nab-paclitaxel, for four cycles. OS and PFS were improved in pembrolizumab plus chemotherapy versus placebo plus chemotherapy (hazard ratio [95% CI], 0.71 [0.59 to 0.85] and 0.62 [0.52 to 0.74]). The 5-year update obtained OS rates of 18.4% versus 9.7%; see Table 1.

Further combinations of tri-modality strategies of chemoradiotherapy (CRT) with IT in clinical trials followed two approaches: IT to boost CRT in locally advanced lung cancer, or RT to boost IT or immuno-chemotherapy in advanced lung cancer.

The first approach occurred in the Pacific Trial [50] for patients with unresectable stage III locally advanced NSCLC treated with concurrent chemoradiotherapy followed by consolidation IT with durvalumab for 12 months for the non-progressive patients, with unexpected good long-term results at five years of 33.1% vs. 19% PFS, 16.9 vs. 5.6 months in median value and 42.9% vs. 33.4% OS, 47.5 vs. 29.1 months, respectively. For patients with PD-L1 expression ≥ 1%, results proved to be even better at 5 years OS, with 50% vs. 36.9%. Due to these unprecedented results, the Pacific regimen became the standard of care in this setting, and a lot of trials followed for less-favorable categories of patients, including patients with sequential CRT, in the Pacific-R [51] or Pacific 6 [52] trials, or even for those with only radiotherapy, to be followed by consolidation durvalumab, in the DUART trial [53], with promising results.

However, up to one third of patients are not eligible for consolidation IT, due to PD, during or after concurrent CRT, radiation pneumonitis, or other adverse events.

A lot of randomized phases II and III trials with CRT and IT followed. A further step was to bring IT into a concurrent setting with CRT. The Keynote 997 phase II trial presented at the American Society of Clinical Oncology (ASCO) meeting in 2021 evidenced that pembrolizumab and concurrent CRT and 14 cycles of consolidation Pembrolizumab present promising antitumor activity, with an overall response rate (ORR) of 70%, regardless of PDL1 and histology, and a manageable safety profile in stage III NSCLC [54]. On the contrary, the final reports of the Pacific 2 trial concluded that starting IT with durvalumab concurrently with CRT, followed by consolidation durvalumab in patients with unresectable stage III NSCLC, did not improve outcomes compared to CRT alone. At a median follow-up of 30.5 months, no significant difference was observed either in PFS (13.8 months compared to 9.4 months, respectively, HR 0.85; 95%CI: 0.65–1.12; *p* = 0.247), nor in OS (36.4 vs. 29.5 months HR 1.03; 95%CI:0.78–1.39; *p* = 0.823). Moreover, in the concurrent durvalumab with CRT arm, PFS and OS were even lower than in the Pacific regimen. Regarding the safety profile in the first months of concurrent treatment, a higher number of adverse events leading to death (13.7% versus 10.2%) or discontinuation (25.6% versus 12.0%) occurred in the durvalumab arm [55]. Also, in the recently reported results of the Checkmate -73L phase III trial [56], simultaneous nivolumab with concurrent CRT followed by consolidation IT with nivolumab, with or without ipilimumab, compared with concurrent CRT and durvalumab consolidation, did not improve PFS versus the Pacific regimen. Therefore, consolidation durvalumab following definitive conc CRT remains the standard of care in this setting.

As intrathoracic recurrence in 80.6% of patients was the main pattern of disease progression in the Pacific study, hypo-fractionated regimens started to be evaluated. A split course of hypo-fractionated RT concurrently with weekly Docetaxel and Cisplatin and 1-year consolidation IT with PD-1/PD-L1 inhibitors was presented at ASTRO 2024, obtaining a better PFS, 25.7 months vs. 16.7 months in the control arm, *p* = 0.044, and absolute lymphocyte count (ALC) proved to be an independent factor in correlation with PFS [57].

IT was evaluated even in neoadjuvant settings in combination therapies. Altkori et al. [58] conducted a phase II trial for early-stage, operable NSCLC with neoadjuvant Durvalumab, alone or combined with immunomodulatory doses of stereotactic radiation of 3 × 8 Gy, obtaining a major pathological response of 6.7% versus 53.3% and, furthermore, a 3-year PFS of 63% compared to 67% in the dual therapy arm.

For metastatic NSCLC, two trials of pembrolizumab, with or without RT, were negative regarding overall response rate, but became positive in the pooled analysis [59]. In the Pembro-RT trial, the first dose of pembrolizumab was given sequentially, less than one week after the last dose of radiotherapy, 24 Gy in three fractions. In the MDACC trial, two regimens of RT were administrated, 50 Gy in 4 fractions, or 45 Gy in 15 fractions, and pembrolizumab was given concurrently with the first dose of RT. The pooled analysis of the two trials encountered 148 patients; 76 received pembrolizumab with RT, 72 pembrolizumab alone. Of the 148 patients, 124 (84%) had non-squamous carcinoma and 111 (75%) received previous CT. The best abscopal response rate was 19.7% with pembrolizumab versus 41.7% with pembrolizumab plus RT (*p* = 0.0039), and the best abscopal control rate was 43.4% with pembrolizumab versus 65.3% with pembrolizumab plus RT (*p* = 0.0071). The median PFS was 4.4 months with pembrolizumab alone versus 9 months with pembrolizumab and RT. The median OS was 8.7 months in the pembrolizumab arm versus 19.2 months for the pembrolizumab combined with RT arm.

**Table 1:** Clinical trials.

Study	Inclusion Criteria	Treatment	Outcomes
Keynote 189 [49]	Adv non-sq cc	Pemetrexed + platinum compound 4 cy ± pembrolizumab → pemetrexed ± pembrolizumab 35 cy	5y-OS: 19.4% vs. 11.3%
The Keynote 407 [19]	Adv sq cc	Paclitaxel or nab-paclitaxel/carboplatin 4 cy ± pembrolizumab 35 cy	5y-OS: 18.4% vs. 9.7%
Pacific [50]	Stage III unr NSCLC	Conc CRT → consolidation durvalumab vs. conc CRT	5y-PFS: 33.1% vs. 19% m PFS: 16.9 vs. 5.6 ms 5y-OS: 42.9% vs. 33.4% m OS: 47.5 vs. 29.1 ms
Pacific R [51]	Stage III unr NSCLC	Conc CRT or seq CRT → consolidation Durvalumab	mrw PFS: 21.7 ms mrw PFS: 23.7 vs. 19.3 ms for conc vs. seq mrw PFS 22.4 vs. 15.6 ms PD-L1 ≥ 1% vs. < 1%
Pacific 6 [52]	Stage III unr NSCLC	seq CRT → consolidation Durvalumab	m PFS of 13.1 ms, m OS of 39.0 ms
Pacific 2 [55]	Stage III unr NSCLC	Conc CRT+ Durvalumab → consolidation Durvalumab vs. conc CTRT	m PFS: 13.8 ms vs. 9.4 ms m OS: 36.4% vs. 29.5%
CheckMate 73L [56]	Stage III unr NSCLC	Conc nivolumab + CRT → nivolumab ± ipilimumab vs. Conc CRT → durvalumab (Pacific Regimen)	no PFS benefit versus the Pacific regimen
Keynote 799 [54]	Stage III unr NSCLC	Conc CRT + pembrolizumab and consolidation pembrolizumab (14 cy)	ORR:70%
Zou et al. Phase I study [57]	Stage III unr NSCLC	Hypo-fractionated RT with Conc CT → consolidation Immunotherapy	m PFS: 25.7 vs. 16.7 ms
Pooled analysis of 2 randomized trials [59]	Adv NSCLC	Pembrolizumab ± conc or seq RT	Best abscopal RR: 19.7% vs. 41.7% m PFS: 4.4 ms vs. 9 ms m OS: 8.7 ms vs. 19.2 ms

Abbreviations. Adv: advanced, non-sq cc: non-squamous carcinoma, cy: cycle, 5-y OS: 5-year overall survival, sq cc: squamous carcinoma, unr: unresectable, conc: concurrent, seq: sequential, CRT: chemoradiotherapy, 5y-PFS: 5-year progression-free survival, m PFS: median progression-free survival, ms: months, m OS: median overall survival, mrw PFS: median real-world progression-free survival, RR: response rate.

## 6. Biomarkers

The most-applied biomarkers of PD-L1 expression, TMB, tumor immune cell infiltrates, and any intrinsic or extrinsic factor affecting them may predict response to IT, as they result from the complex mechanisms of resistance described; see Table 2 [14].

Tumor immune cell infiltrates evidently predict better response in patients receiving IT, than in cases with tumors characterized by immune desert. Even more available, the absolute lymphocyte count (ALC) has been reported to predict RT outcomes and abscopal effect [44,60,61,62]. In an analysis of 165 patients from three prospective trials, evaluating a combination of RT and IT found that pre-RT ALC was correlated with significantly better PFS regardless of stereotactic or traditional RT. The data from three institutional phase 1/2 trials reported that for post-RT ALC higher than the median value, the abscopal response rate was 34.2% vs. 3.9% in patients with lower than the median value. In the experience of “Ion Chiricuta Oncology Institute”, using different anti-PD-1 checkpoint combination strategies for first-line advanced NSCLC treatment, the 4-year survival was significantly higher, 32.3% vs. 8.2%, in patients with neutrophils to lymphocytes ratio (NLR) ≤ 3.81 than in those with NLR > 3.81, *p* < 0.01. Older age, impaired performance status (PS), corticotherapy in the first month of IT, and NLR > 3.81 were independent unfavorable prognostic factors in the multivariate analysis of survival [63]. A meta-analysis including one hundred studies comprising 40,559 patients with different solid tumors found a median cutoff for NLR of 4. Values greater than the cutoff predicted shorter PFS and OS; HR for PFS was 1.63 and for OS was 1.81 (95% CI = 1.67 to 1.97; *p* < 0.001) [64].

High PD-L1 expression in tumor cells is generally associated with better response to PD-1/PD-L1 inhibitors in NSCLC patients. PD-L1 expression ≥ 1% was correlated with improved survival in the combined long-term results of the CheckMate 017 and CheckMate 057 trials [7,8,9,10], or after CRT in the Pacific trial [50]. But in the Keynote 189 and 407 trials, IT combined with CT gave better outcomes, regardless of PD-L1 status [48,49].

High TMB assumes tumor-specific neoantigens that contribute to the immune recognition of tumor cells and is associated with better outcomes to ICIs; however, tumors with HLA loss behave like tumors with low TMB. However, obtaining information by whole exome sequencing using tumor biopsy samples is hardly achievable in everyday clinical practice. Moreover, non-invasive strategies like blood-based assays using circulating tumor DNA (ctDNA) are of great potential but under investigation [65].

As shown above, driver mutations in NSCLC patients proved limited benefit from ICB. However, high PD-L1 expression was found in 19–20% of cases with classic EGFR, EGFR exon 20, and HER2 mutant tumors and 34–55% in tumors with ALK, BRAF, ROS, RET, or MET alterations [22].

Despite works that have shown an increase in PD-L1 expression via IL-6/JAK/STAT3 [66] or p-ERK1/2/p-c-Jun signaling but not through the p-AKT/p-S6 pathway [67], Biton et al. found that EGFR-mutated NSCLC cells were found to have a lower PD-L1 expression and weaker immunogenic TME [68].

KRAS alterations are the most frequent oncogenic driver mutations in NSCLC, but characterized by a phenotypic heterogeneity, and a higher TMB has been observed [68,69].

BRAF mutations were associated with greater clinical benefit from ICB, which may be attributed to higher TMB and PD-L1 expression in tumor cells [22].

RET and HER2 mutations attenuated PD-L1 expression, while ALK, ROS, and MET enhanced PD-L1 expression but downregulated TMB and TILs, leading to resistance to ICIs [14,22]. However, EGFR/HER2 mutations and ALK, ROS, RET, and MET fusions defined NSCLC subsets with minimal benefit from ICB, despite high PDL1 expression indicating that PD-L1 expression and TMB seem to have an independent impact on sensitivity to IT. This requires further investigations regarding the intrinsic or extrinsic factors affecting them, in the complex mechanisms of resistance involved, to predict potential therapeutic strategies [22].

Chen et al. found that the apolipoprotein B mRNA editing enzyme catalytic polypeptide-like (APOBEC) signature mutational activity is correlated with high TMB and PD-L1, LAG3 immune checkpoint associated gene markers, and CD8+ and CD4+ T immune cell infiltration markers. Furthermore, the APOBEC gene family is correlated with IL2-STAT5 gene involvement in the INF gamma signaling pathway. Individual gene mutations IFNGR1 or VTCN1 were also found to be associated with response, whereas PTEN was associated with resistance to IT [70].

Also, Xu et al. revealed that patients with NFE2L2/KEAP1 gene mutation were correlated with higher TMB and PD-L1 expression, improving clinical outcome to IT [71].

Recent studies have shown that mutations in KRAS, SKT11, KEAP1, JAK1/2, B2M, APC, MTOR and TP53 and co-mutations of these genes are the main determinants of ICB response in non-small-cell lung cancer (NSCLC) patients, influencing metabolic changes in cancer cells, cytotoxic T cells, and the efficacy of ICIs [26,27].

TP53 mutation with STK11/EGFR WT identified a tumor-immune profile by enriched expression of PD-L1 and CD8+ T cells in the TME, by the upregulation of chemokines CXCL9, 10, 11, and a stronger expression of genes involved in antigen processing and MHC-1 presentation, indicating a higher immunogenicity.

On the contrary, combinations of TP53 and STK11 alterations were associated with lower PD-L1 expression and immune cell infiltration, indicating that the effect of STK11 mutation dominates over TP53 mutation. [68]. Dong et al. using gene set enrichment analysis to determine potentially relevant gene expression and predict response to ICB, and found that co-mutant TP53 and KRAS were responsible for enhanced PD-L1 expression, TMB, and CD8+Tcell infiltration [69].

Resistance mutations were identified in 27.8% of patients who received IT and included acquired loss-of-function mutations in STK11, B2M, APC, MTOR, KEAP1, and JAK1/2. These acquired alterations were not observed in the control groups. Immunophenotyping of matched pre- and post-ICI samples demonstrated significant decreases in intratumoral lymphocytes, CD3e+ and CD8a+ T cells, and PD-L1/PD-1 engagement, as well as increased distance between tumor cells and CD8+ PD-1+ T cells. There was a significant decrease in HLA class I expression in the IT cohort at the time of acquired resistance compared with the control groups receiving CT (*p* = 0.005) and targeted therapy (*p* = 0.01) cohorts [27]. Furthermore, Ricciuti et al. [72] analyzed clinical outcomes to PD-1/PD-L1 inhibition according to KRAS, STK11, and KEAP1 mutation status in two independent cohorts, at the Dana-Farber Cancer Institute/Massachusetts General Hospital cohort and the Memorial Sloan Kettering Cancer Center/MD Anderson Cancer Center cohort, and found that STK11 and KEAP1 mutations confer worse outcomes to IT among patients with KRAS mutant but not KRAS wild type lung adenocarcinoma. Tumors harboring co-occurring genomic alterations in STK11 or KEAP1 genes to KRAS mutations define a distinct immune profile in terms of gene expression and immune cell infiltration.

The STK11 gene regulates diverse cellular metabolic functions. STK11 loss occurs in approximately 15% of lung adenocarcinomas and is associated with a lack of PD-L1 expression, reduced tumor-infiltrating cytotoxic CD8+ T lymphocytes, and resistance to ICI in patients with KRAS mutant NSCLC. KEAP1 loss occurs in approximately 20% of NSCLC cases and is associated with an immunosuppressive microenvironment characterized by low infiltration of CD8+ T cells [72].

Zielinski et al. also correlated resistance mechanisms with genetic mutation like STK11, KEAP1, JAK1/2. Moreover, STK11 mutations, besides M2 macrophage polarization and T cell dysfunction, produce epigenetic changes responsible for metabolic alterations like methylation, histone acetylation, and increased lactate production. KEAP1 is responsible for increased consumption of glucose and glutamine by tumor cells, producing deprivation of nutrients of the T cells. JAK1/2 mutation disrupts interferon–gamma signaling, responsible for DC activation [73], suggesting therapeutic strategies to correct this metabolic alteration.

Additional aspects are connected to the extracellular matrix (ECM), the non-cellular scaffold around cells, which acts as a physical barrier to immune cell infiltration, shapes the TME, and sends biochemical signals that suppress or activate immune responses. The various components of ECM, like collagens, hyaluronan, and matrix metalloproteinases, are responsible for poor immune cell infiltration, exclusion, and altered immune cell trafficking, respectively. Proteoglycans like versican can inhibit the functions of dendritic cells and T cells. High fibronectin expression correlates with an immune-suppressive microenvironment and reduced response to immunotherapy. Consequently, these components or fragments are potential biomarkers valuable to investigation in clinical practice [74].

**Table 2:** Biomarkers.

Biomarker	Outcomes	Observations	Studies/ References
NLR	NLR < 3.81: 4y-S 32.3%	NLR > 3.81 independent unfavorable prognostic factors	[63]
	NLR > 4: Shorter PFS, OS [64]	Median cutoff for NLR of 4, in a meta-analysis for patients with different solid tumors	[64]
PD-L1 expression	PD-L1 ≥ 1% 5y-OS 18.8% vs. 3.4%	vs. all patients	[10]
	PD-L1 ≥ 1% 5y-OS 50% vs. 42.9%	vs. all patients	[50]
High TMB	Benefit from ICB	Tumors with HLA loss behave like tumors with low TMB	[27,65]
Circulating tumor DNA	Great potential	Under investigation	[65]
**Driver mutations and alterations**	Limited benefit from ICB		[22]
EGFR, HER2 genes	Lower PD-L1 expression, and TME	Controversial results	[68]
	PD-L1 expression in 19–20% of cases, low TMB	PD-L1 expression and TMB have independent impact on sensitivity to IT	[22]
ALK, ROS, RET, MET alterations	PD-L1expression in 34–55% of cases, low TMB	PD-L1 expression and TMB have independent impact on sensitivity to IT	[22]
KRAS gene	Higher TMB	The most frequent oncogenic driver mutations in NSCLC, but characterized by a phenotypic heterogeneity	[68,69]
BRAF gene	Higher PDL1 and TMB	Greater benefit of ICB	[22]
**Individual gene mutations**			
IFNGR1 gene	Benefit from ICB		[70]
VTCN1 gene	Benefit from ICB		[70]
PTEN gene	Resistance to ICB		[70]
APOBEC signature	High PD-L1, TMB and TILs	Associated with LAG3 and STAT5 gene involvement	[70]
**Co-mutation genes**			
TP53+KRAS	Enhanced PD-L1 expression +TMB+ CD8+ T cell infiltration	Benefit from ICB	[69]
TP53+STK11/EGFR WT	Enhanced PD-L1 expression + CD8+ T cell infiltration	Benefit from ICB	[68]
TP53+STK11 alterations	Lower PD-L1 expression and TILs	Resistance to ICB; STK11mutation dominates over TP53 mutation	[68]
**Resistance gene mutations**	Resistance to ICB	Observed in 27% of patients receiving IT, but not in control groups	[72,73]
STK11, B2M, APC, MTOR, KEAP1, and JAK1/2	Decrease in PD-L1, HLA class I expression, TILs	Worse outcome in STK11 and KEAP1 mutations combined with KRAS mutation but not with KRAS WT	[72]
STK11 in 15% of adcc	Low PD-L1 expression and TILs	Epigenetic changes responsible for metabolic alterations: methylation, histone acetylation, and increased lactate production	[72,73]
KEAP1 in 20% of NSCLC	Immunosuppressive TME	Increased consumption of glucose and glutamine by tumor cells	[72,73]
JAK1/2 mutation	Disrupts INF-signaling responsible for DC activation		[73]

Abbreviations: NLR: neutrophils to lymphocytes ratio, OS: overall survival, PFS: progression-free survival, PD-L1: TMB: tumor mutational burden, ICB: immune checkpoint blockade, DC: dendritic cell, HLA: Human Leukocyte Antigen, TILs: Tumor infiltrating lymphocytes, TME: tumor microenvironment, INF: interferon.

## 7. Future Directions

### 7.1. New Actionable Checkpoints

As only a percentage of cases respond to CTLA4, PD-1-PD-L1 axis blocking, multiple agents are in development to address to new actionable checkpoints, like TIGIT, LAG3, TIM3, NKG2A, and CD-73 [44,73,75,76,77].

The T cell Immune-receptor with immune-globulin and ITIM domain (TIGIT) is a coinhibitory immune-modulatory checkpoint receptor, present on T cells CD8+, CD4+, T regs, and NK cells, which binds to ligands CD112 and CD115 on tumor cells and APCs.

The costimulatory receptor CD226 competes with TIGIT for binding ligands CD112 and CD115, restoring immune antitumor response. In the Cityscape trial, the combination of tiragolumab (an anti TIGIT Ab) + atezolizumab vs. atezolizumab in PD-L1 positive, recurrent, or metastatic NSCLC, in first-line treatment, obtained a response rate of 31% vs. 16%, m PFS of 5.4 months vs. 3.6 months, HR 0.57, *p* = 0.015 [75]. Serious treatment-related adverse events were 21% vs. 18%.

Lymphocyte activation gene-3 (LAG-3) is an inhibitory molecule on CD8+, CD4+ T cells and other cells. Increased expression and co-expression of PD-1 results in a greater T cell dysfunction and is associated with resistance to PD-1 blockade. The Relativity-104 phase II trial, presented at ESMO 2024, evaluated relatlimab, a human LAG-3 blocking Ab with nivolumab and platinum doublet CT vs. nivolumab and platinum doublet CT, obtaining benefits in median duration of response and PFS, HR 0.88, especially in prespecified groups like PD-L1 > 1% (m PFS 9.8 vs. 6.1 months, HR 0.63) with manageable safety profile [76]. Trials with relatlimab and even bispecific LAG-3 and PD-1 antibodies are ongoing.

Other receptors expressed on NK cells, DCs, monocytes, and macrophages responsible for immune tolerance are T cell immunoglobulin and mucin domain containing protein 3 (TIM-3); Natural killer group protein 2A (NKG-2A), which triggers immune suppression; and CD-73 overexpressed on TAMs, T regs, and exhausted T cells, responsible for TME suppression [77].

Also, there are additional inhibitory checkpoints on T cells, APCs, and tumor cells under investigation.

### 7.2. Novel Types of Immunotherapies

Moving beyond checkpoint inhibitors, novel types of IT emerge like STING agonists, which stimulate downstream production of the Type I interferon, responsible for the activation of DCs, amongst other immunostimulatory events, and CD40 agonists, which enhance DC function, stimulate T cell trafficking, and activate M1 polarized macrophages and Toll-like Receptor agonists that activate T cells and convert MDSC into immunostimulatory antigen-presenting cells [44]. These agonists might be therapeutic strategies in JAK1/2 mutations which disrupt interferon–gamma signaling. In STK11 and KEAP1 mutations, corrections of the metabolic changes, like DNA methyltransferase inhibitors or histone deacetylase inhibitors, respectively, glutaminase inhibitors can be associated with IT.

## 8. Conclusions and Perspectives

Many studies on combination therapies such as chemo-radio-immunotherapy are ongoing, and results are awaited. Taking into account the clinical considerations affecting tumor immunogenicity, further investigations are required to study RT dose/fractionations and the effects on TME, RT volume, sites (primary tumor, lymph nodes or metastases), and sequencing and timing of the multimodality treatments.

The genomic and immunophenotypic heterogeneity of resistance to IT in NSCLC will need to be considered when developing novel therapeutic strategies aimed to overcome resistance.

Further studies need to investigate multiple agents addressing coinhibitory receptors and costimulatory receptors, and multiple additional inhibitory targets on T cells, APCs, and tumor cells.

The huge potential of combination therapies is becoming apparent. Questions regarding targets, selection of patients, and time and sequence of administration are yet to be answered, considering the complex mechanisms of resistance. Dynamic biomarkers are needed to guide more personalized treatment decisions.

## Data Availability

Not applicable.
